# A Herbal Composition of *Scutellaria baicalensis* and *Eleutherococcus senticosus* Shows Potent Anti-Inflammatory Effects in an Ex Vivo Human Mucosal Tissue Model

**DOI:** 10.1155/2012/673145

**Published:** 2012-01-05

**Authors:** Nan Zhang, Koen Van Crombruggen, Gabriele Holtappels, Claus Bachert

**Affiliations:** ^1^Department of Oto-Rhino-Laryngology, Upper Airway Research Laboratory (URL), Ghent University Hospital, De Pintelaan 185, 9000 Ghent, Belgium; ^2^Department of Oto-Rhino-Laryngology, Zhongshan City Peoples's Hospital, Zhongshan 528403, China

## Abstract

*Background*. Patients seek an effective alternative to pharmacotherapy including herbal treatment options for allergic rhinitis and rhinosinusitis. *Material and Methods*. Nasal mucosal tissue was obtained from 12 patients, fragmented, preincubated with tissue culture medium, *S. baicalensis* and/or *E. senticosus* and/or vitamin C (each compound 0.2 **μ**g/mL and 2 **μ**g/mL) for 1 hour at 37°C/5% CO2, and stimulated with anti-IgE for 30 minutes and 6 hours to imitate the allergic early and late phases. Furthermore, Staphylococcus aureus superantigen B (SEB) stimulation for 6 hours was used to imitate T-cell activation. *Results*. The combination of *S. baicalensis* and *E. senticosus* had a more potent suppressive effect on the release of PGD2, histamine, and IL-5 than *S. baicalensis* alone. The combination also resulted in a significant inhibition of SEB-induced cytokines comparable or superior to an established topical corticosteroid, fluticasone propionate. Vitamin C increased ciliary beat frequency, but had no anti-inflammatory effects. *Discussion*. The combination of *S. baicalensis* and *E. senticosus* may be able to significantly block allergic early-and late-phase mediators and substantially suppress the release of proinflammatory, and Th1-, Th2-, and Th17—derived cytokines.

## 1. Introduction

Chronic upper airway diseases such as allergic rhinitis and chronic rhinosinusitis are common and known to be disabling diseases, and respectively account for 20% and 11%, of the European adult population [1, 2]. Treatment options have been summarized in recent guidelines [3, 4], and evidence-based recommendations have been formulated. The recommended pharmacologic approaches include antihistamines and topical corticosteroids as first choice treatments. However, patients may reject those treatment options due to common side effects, lack of efficacy, and a phobia for steroid use. An increasing number of patients seek an efficacious alternative, including herbal treatment options [5].

Allergic rhinitis (AR) represents an inflammation of the nasal mucosa to allergens after a sensitization process. Typically, symptoms occur rapidly after allergen exposure; sneezing, and itching manifest within seconds or a few minutes, nasal secretion and obstruction following within 30 minutes. A second so-called late-phase response with nasal congestion as the main symptom has been demonstrated, which is paralleled by the influx of eosinophils [3]. Mast cells are pivotal in the immediate response, releasing histamine among other mediators such as leucotrienes and prostaglandins, and Th2 cells are known to orchestrate the late phase response via the release of potent cytokines including interleukin IL-4, IL-5, and IL-13.

Also acute postviral (ARS) and chronic rhinosinusitis with or without nasal polyps (CRSsP, CRSwNP) are currently understood to represent an inflammatory reaction, orchestrated by either Th1, Th2, or Th17 cells, consequently involving other cell types such as neutrophilic or eosinophilic granulocytes [4]. Although the resulting tissue environment may be clearly different between those inflammatory reactions, they do share similarities such as the involvement of proinflammatory cytokines formed within the mucosa.

The above elicited pathophysiologic responses are best observed in the human subject as is their response to specific therapies aimed to treat the underlying pathogen. However, for purposes of medicinal evaluations and showing the effect of new therapeutic agents a less invasive and risk-free model is needed to show effective response. Towards that end, we developed ex vivo mucosal tissue models using human nasal mucosa freshly harvested during routine nasal and sinus surgery [6, 7]. These models have been specifically stipulated to be used for allergic conditions making use of leucotriene de novo synthesis of mast cells, or T helper cell biased inflammatory conditions measuring cytokines derived from different T helper cells as outcome parameters. Different stimuli have been identified for the various conditions [6, 8]. Furthermore, standard drugs currently used in such conditions (e.g., topical corticosteroids in allergic rhinitis and acute or chronic rhinosinusitis) with a known response can be used to compare the newly tested therapy facilitating the assessment of effect and the therapeutic potential of the “new arrival”.

Over the last two decades interest has mounted regarding the mechanism of action of herbal therapies [5, 9]. Many attempts to identify the active components of herbal remedies have concluded that in general no one component is responsible for the therapeutic capacity, but rather a complex and intricate interaction of various herbs may result in therapeutic efficacy. Additionally, many of these herbal combinations have begun to demonstrate anti-inflammatory actions. Although the majority of herbal medications are delivered orally, topical applications have also been practiced.

We researched the literature for herbal compounds that have inhibitory effect on the release of mediators and cytokines typically involved in upper airway disease such as allergic rhinitis and rhinosinusitis. Of the various herbal entities considered, *Scutellaria baicalensis* and *Eleutherococcus senticosus* have been reported to elicit effects on inflammation, especially of allergic origin [10–13].

In an environmentally induced inflammation model, *S. baicalensis* had significant anti-inflammatory effects by inhibiting the NF-*κ*B pathway [14]; these observations were recently extended to IKK*αβ* and I*κ*-B*α*, further supporting the potent anti-inflammatory activity through the MAP kinase pathway [15]. In skin-inflammation models (atopic dermatitis), *S. baicalensis* modulated immunological responses mainly through influencing IL-5 or IL-10 levels [16], inhibited prostaglandin E2 generation, and suppressed the expression of proinflammatory genes, cyclooxygenase-2, and interleulin-1beta in the skin lesion [17]. Also, *E. senticosus* inhibited mast cell-mediated anaphylaxis in a murine model [18] and significantly attenuated LPS-induced iNOS expression by blocking transcription pathways in macrophages [19].

After screening these herbal compounds, we created a formula which includes *S. baicalensis*, *E. senticosus,* and vitamin C. This combination demonstrated a reproducible and meaningful effect on mast cell and T cell mediators.

Our objective was to show the anti-inflammatory effect of this herbal/vitamin formulation in comparison to fluticasone propionate, a well-established topical corticosteroid for the treatment of upper airway inflammation [6], in our ex vivo human mucosal model.

## 2. Materials and Methods

### 2.1. Patients

Nasal mucosal tissue was obtained from a total of 12 patients (mean age 44 years, range 22 to 67 years) during routine sinus surgery, who either had a septal deviation and conchal hypertrophy resulting in nasal obstruction (*n* = 6 for inferior turbinate tissue, used for mast cell related experiments) or nasal polyps (*n* = 6 for polyp tissue, used for mast cell-and T-cell-related experiments), at the Department of Otorhinolaryngology of the University Hospital of Ghent. The ethical committee of the Ghent University Hospital approved the study, and all patients gave their written informed consent prior to inclusion in the study.

None of the subjects received intranasal corticosteroids, anti-histamines, anti-leukotrienes, oral or intranasal decongestants, or intranasal anticholinergics within the 2 weeks prior to surgery. None of the subjects received oral and/or intramuscular corticosteroids within the 4 weeks prior to surgery. The female subjects who were pregnant or breast-feeding were excluded from the study. Nasal polyps were diagnosed based on symptoms, clinical examination, nasal endoscopy, and sinus computed tomography (CT) scan according to the EP3OS guidelines [3]. Inferior turbinate hypertrophy was diagnosed by nasal endoscopy. Patients were included independent of their atopic status.

### 2.2. Mechanical Disruption and Stimulations of Human Nasal Tissue

The human nasal mucosa and submucosa was cut thoroughly in tissue culture medium consisting of RPMI 1640 (Sigma-Aldrich, Bornem, Belgium), containing 2 mM LGlutamine (Invitrogen, Merelbeke, Belgium), antibiotics (50 IU/mL penicillin and 50 *μ*g/mL streptomycin) (Invitrogen), and 0.1% BSA (Bovine Serum Albumin, Sigma).

The tissue was passed through a mesh to achieve comparable fragments. The tissue fragments (±0.9 mm^3^) were weighed and resuspended as 0.04 g tissue/1 mL tissue culture medium and preincubated with tissue culture medium, *S. baicalensis* and/or *E. senticosus* and/or vitamin C (each compound 0.2 *μ*g/mL and 2 *μ*g/mL) for 1 hour at 37°C/5% CO2. For anti-IgE stimulations, the tissue was preincubated for 1 hour at 37°C, 5% CO2 with 1 *μ*g/mL human myeloma IgE (Calbiochem, VWR International, Leuven, Belgium). After 3 washing steps the tissue fragments were resuspended in the appropriate amount of culture medium, and 0.5 mL of this fragment suspension was dispensed per well of a 48-well plate. (BD Falcon, VWR, Leuven, Belgium).

### 2.3. Models for Mast Cell Degranulation and T-Cell Activation

The models for mast cell degranulation and T-cell activation were used as described before [6–8]. For mast cell-related stimulations, the fragment suspensions were stimulated with either culture medium (negative control) or *ε*-chain specific anti-human IgE antibody (Dako Belgium N.V., Heverlee, Belgium) at 10 *μ*g/mL (Dako Belgium N.V., Heverlee, Belgium) for 30 minutes. Supernatants were separated by centrifugation and stored immediately at −20°C until analysis of histamine and PGD2. Concentrations of histamine and PGD2 were measured in tissue supernatants obtained after the stimulations using ELISA kits for Histamine (IBL Hamburg, Germany; detection limit 2.7 ng/mL) and PGD2 (Cayman Chemicals, Ann Arbor, Michigan; detection limit 2 pg/mL) following the instructions of the manufacturer.

For T-cell-related stimulations, tissue fragments were stimulated with culture medium (negative control), 0.5 mg/mL staphylococcus aureus enterotoxin B (SEB, Sigma-Aldrich), or *ε*-chain-specific anti-human IgE antibody (Dako Belgium N.V., Heverlee, Belgium) at 10 *μ*g/mL (Dako Belgium N.V., Heverlee, Belgium) for 6 hours. Supernatants were separated by centrifugation; aliquots of the supernatants were stored immediately at −20°C until analysis of cytokines. Concentrations of IL-1*β*, TNF-*α*, IFN-*γ*, IL-2, IL-5, and IL-17 (detection limits 0.6 to 7.8 pg/mL) were measured in tissue supernatants obtained 6 h after the ex vivo stimulations using commercially available ELISA kits (Quantikine ELISA, R&D Systems, Minneapolis, Minn) following the instructions of the manufacturer. The topical glucocorticosteroid fluticasone propionate was used as a positive control in concentrations of 10^−10^ to 10^−8^ molar for all stimulations with SEB. Tissues were incubated with either single compounds or the final combination of herbs for 1 hour prior to stimulations.

### 2.4. Toxicity and Ciliary Beat Frequency

Peripheral blood mononuclear cells (PBMCs) consisting mainly of monocytes, T cells, and B cells and smaller amounts of NK cells and dendritic cells of both myeloid and plasmacytoid origin of 3 donors were used for the assessment of potential toxic risks of the used compounds. PBMCs were incubated in the presence or absence of compounds, either single or in combination, and cell viability was analysed by trypan blue exclusion.

Preservation of ciliary function was tested utilizing the Sissons Ammons Video Analysis (SAVA) system; we evaluated the acute effects of the herbal preparations at different concentrations on freshly harvested mouse nasal septa. Baseline cilia beat frequency was obtained for 10 minutes prior to application of the herbal extracts. Cilia beat frequency was continuously monitored for an additional 15 minutes following application of the test substances.

### 2.5. Statistical Analysis

Statistical analysis was performed using the Wilcoxon test (for paired comparisons). The Mann-Whitney *U* test was used for between-group (unpaired) comparisons. *P* values of less than 0.05 were considered statistically significant.

## 3. Results

### 3.1. Mast Cell-Related Mediators: Prostaglandin D2 and Histamine (Figure 1)

Anti-IgE resulted in a significant increase in prostaglandin D2 (PGD2) and histamine release within 30 minutes of tissue exposure in both inferior turbinate and nasal polyp mucosal tissue. In comparison to tissue culture medium, *S. baicalensis*, but not *E. senticosus*, significantly reduced the release of de novo synthesized PGD2 at a concentration of 20 *μ*g/mL in inferior turbinates and 2 *μ*g/mL in nasal polyps. *S. baicalensis* at this concentration also reduced the release of histamine in stimulated nasal polyps.

**Figure 1:**
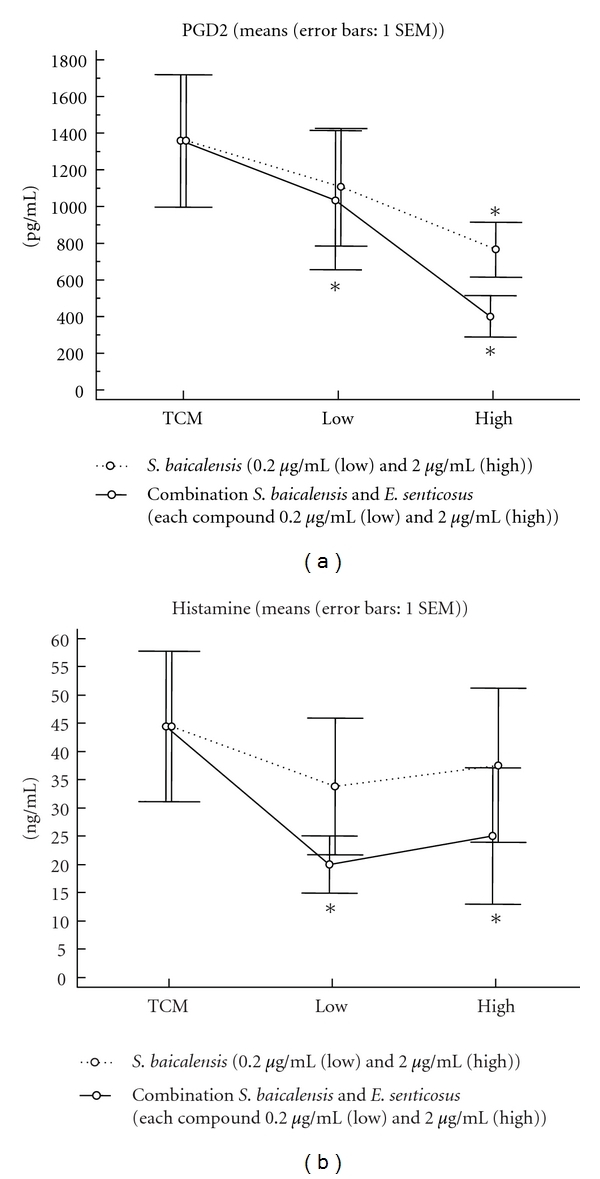
Mast cell-related mediators: Prostaglandin D2 (a) and histamine (b). Anti-IgE resulted in a significant increase in prostaglandin D2 (PGD2) and histamine release within 30 minutes after tissue exposure in nasal polyp mucosal tissue. In comparison to tissue culture medium, *S. baicalensis*, but not *E. senticosus* (data not shown), significantly reduced the release of de novo synthesized PGD2 at a concentration of 2 *μ*g/mL (high) in nasal polyps. The combination of *S. baicalensis* and *E. senticosus*, however, had a more potent effect on the release of PGD2 in nasal polyp tissue and reached significance already at a low concentration of 0,2 *μ*g/mL of each compound after anti-IgE stimulation (a). The combination of *S. baicalensis* and *E. senticosus* had a similarly strong suppressive effect at a low concentration of 0.2 *μ*g/mL to reduce the release of histamine (b).

The combination of *S. baicalensis* and *E. senticosus*, however, had a more potent effect on the release of PGD2 in inferior turbinate tissue (significance reached for 2 *μ*g/mL for each component) and nasal polyps (1 *μ*g/mL); a concentration of 0.2 *μ*g/mL totally suppressed the release of PGD2 from nasal polyp tissue after anti-IgE stimulation (Figure 1(a)).

Similarly, *S. baicalensis*, but not *E. senticosus*, induced a significant and nearly complete suppression of anti-IgE-induced histamine release in nasal polyps at a concentration of 20 *μ*g/mL. The combination of *S. baicalensis* and *E. senticosus*, however, had a similarly strong suppressive effect on histamine release with only 0.2 *μ*g/mL of each compound (Figure 1(b)).

Vitamin C alone or in combination with *S. baicalensis* or *E. senticosus* did not show any additional significant effect on the release of PGD2.

### 3.2. Anti-IgE Induced Cytokine Release (Figure 2)

Anti-IgE stimulation of nasal polyps resulted in an increase in IL-5 within 6 hours; this release could significantly be suppressed by corticosteroid pretreatment. Furthermore, the combination of *S. baicalensis* and *E. senticosus*, but no single component, resulted in a significant inhibition of IL-5 release at a concentration of 2 *μ*g/mL of each component.

### 3.3. T-Cell Activation-Related Release of Cytokines (Figure 3)

Spontaneous release of IL-5, and TNF-*α* could be observed in nasal polyp tissue, which was significantly inhibited by the combination of *S. baicalensis* and *E. senticosus*, but no single component, at a concentration of 2 *μ*g/mL of each component (data not shown).

**Figure 3:**
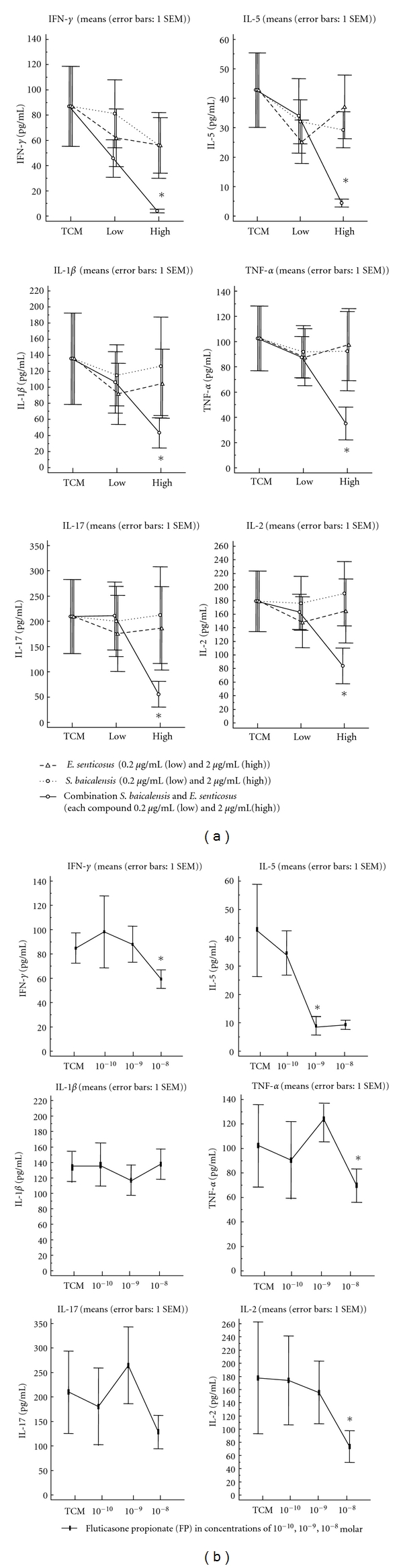
T-cell activation-related release of cytokines. (a) SEB resulted in a significant release of IL-1, TNF-*α*, IFN-*γ*, IL-2, IL-5, and IL-17 from nasal polyp tissue, indicating the stimulation of all prominent T helper cell populations. Although single components demonstrated suppressive activity, this suppression remained insignificant; only the combination of *S. baicalensis* and *E. senticosus* at a concentration of 2 *μ*g/mL of each compound resulted in a significant, nearly complete suppression of the release of IFN, IL-5, IL-1*β* and TNF-*α* 6 h after SEB stimulation, and a partial suppression for IL-17 and IL-2. (b) The suppressive activity of the combination was superior to that of an established topical corticosteroid, fluticasone propionate (FP), for IFN-*γ*, IL-1*β*, TNF-*α*, and IL-17 and equivalent for IL-5 and IL-2.

SEB resulted in a significant release of IL-1, TNF-*α*, IFN-*γ*, Il-2, IL-5 and IL-17 from nasal polyp tissue, indicating the stimulation of all prominent T helper cell populations. Although single components demonstrated suppressive activity, this suppression remained insignificant; only the combination of *S. baicalensis* and *E. senticosus* at a concentration of 2 *μ*g/mL of each component resulted in a significant, nearly complete suppression of the release of IFN, IL-5, IL-1*β*, and TNF-*α* 6 h after SEB-release, and a partial suppression for IL-17 and IL-2 (Figure 3(a)). The suppressive activity was superior to that of an established topical corticosteroid, fluticasone propionate, for IFN-*γ*, IL-1*β*, TNF-*α*, and IL-17 and equivalent for IL-5 and IL-2 (Figure 3(b)).

Vitamin C alone or in combination with *S. baicalensis* or *E. senticosus* had no significant effect on the release of cytokines in this model.

### 3.4. Toxicity, Ciliary Beat Frequency

The pH assessments showed normal, physiological values for all dilutions used. There was no toxicity of the compounds, either single or in combination, at concentrations lower than or equal to 10 *μ*g/mL. SEB as well as higher concentrations of the mix induced a mild toxicity (less than 15% of cells were apoptotic).

Application of the highest concentration of the combination of *S. baicalensis* and *E. senticosus* did not affect ciliary beat frequency. However, vitamin C significantly increased ciliary beat frequency from a concentration of 0.1%. This effect was not different when vitamin C was applied in combination with the herbal compounds.

## 4. Discussion

We here present a human nasal mucosal model which allows testing of drug compounds in several aspects; we demonstrated the ability of a herbal composition to inhibit the release of allergic immediate and late-phase mediators as well as the release induced by the activation of different T cells. We were able to establish that preincubation of *S. baicalensis* for one hour is able to significantly suppress the release of histamine and PGD2 upon anti-IgE stimulation of mucosal tissue and that the combination of *S. baicalensis* with *E. senticosus* and vitamin C increases the suppressive efficacy of PGD2 and histamine. This combination was shown to lower the concentrations and nearly completely suppress both PGD2 and histamine.

In line with these findings, within 6 hours after stimulation, anti-IgE-induced release of IL-5 was significantly suppressed by the herbal combination. Furthermore, the herbal combination, but no single component on its own, was able to inhibit a wide range of T effector cells to release specific cytokines upon SEB stimulation, including Th1, Th2 and Th17 cells. This effect was partially superior, but certainly equivalent to the activity of topical corticosteroids in terms of the range and the degree of suppression of cytokines.

Stimulations with anti-IgE on IgE-primed nasal tissue fragments lead to a concentration-dependent release of histamine, leukotrienes, and PGD2 in our mucosal model, as was described earlier [7]. The release of these early-phase mediators was significantly higher in nasal polyps compared to inferior turbinates, although tryptase, Fc epsilon RI alpha positive cells, and Fc epsilon RI alpha-chain transcripts were equally present in both groups. Thus, nasal polyp tissue would be especially suitable for tissue stimulation assays, with a more robust read-out allowing comparative studies using different compounds. We tested different tissues, inferior turbinate, and nasal polyp mucosa and showed similar results, confirming the findings. Again, the finding of a suppression of PGD2 and histamine release points to a mast cell stabilizing and not just de novo synthesis of PGD2-inhibiting effect of *S. baicalensis* as single compound, but also as the most effective component in the herbal combination.

In an independent set of studies, we demonstrated that a Syk inhibitor prevented mast cell degranulation assessed by the measurement of histamine release and the production of PGD2 [8]. Furthermore, the Syk inhibitor was similarly able to significantly inhibit the release of these granules and newly synthesized mediators by nasal polyp mast cells in a dose-dependent manner. These studies show the robustness of the mucosal tissue assays and their usefulness to test mast cell-related therapeutic approaches in human tissue ex vivo.

The release of IL-5 several hours after anti-IgE stimulation has been described as part of the late-phase allergic reaction, related to the influx and activation of eosinophilic granulocytes, for which IL-5 is a key survival and proliferation factor [3, 20]. The combination of these effects—suppression of early-and late-phase allergic mediators—by one herbal composition may be of great relevance in the treatment of allergic diseases such as allergic rhinitis, as it would target sneezing and secretion as well as nasal obstruction typically developing over minutes and hours upon exposure to the allergen; such an approach would have a fast onset of action by suppressing the release of histamine, and at the same time a high anti-inflammatory efficacy by inhibition of the allergic eosinophilic response.

Apart from mast cell-derived cell products, the herbal composition also significantly inhibited the SEB-induced activation of different T-cell subsets. In former studies we have shown that the activation of T cells by SEB is a relevant naturally occurring event in the airways (refs), resulting in the activation of Th1, Th2, Th17, and other cells and that nasal polyp tissue is a suitable model tissue for exposure tests, as they comprise a vast range of different T cell subsets [6]. T-cells orchestrate immune defence and inflammation; currently, 3 major T effector cells are described (Th1, Th2, and Th17 cells). These cells are important in different forms of inflammation and substantially impact the composition of inflammatory cells (eosinophilic versus neutrophilic), and also induce pathogenic changes such as mucus production and remodelling [21]. Th2, Th1, and Th17 cells have been implicated in chronic diseases of the paranasal sinuses, that is, Th1-related cytokine IFN-*γ* in chronic rhinosinusitis without nasal polyps (CRSsNP), Th2-related cytokines IL-4 and IL-5 in CRS with nasal polyps (CRSwNP), and recently Th17-cell-related IL-17 in Asian nasal polyps and polyps in cystic fibrosis patients [22, 23]. As those cytokines may also present within the same tissue, a drug with a broad range of activities on different T cells is needed to efficiently downregulate inflammation.

Recent findings demonstrate that the specific activation of the inflammatory pathway by superantigens such as SEB results in a severe local inflammation and the systemic spread of disease, such as asthma in nasal polyp patients [24–26]. Although topical corticosteroids are a mainstay for the treatment of airway inflammation, and nasal polyps specifically, they do have merits—the nearly complete suppression of Th2 cells—and deficits in terms of an incomplete suppression of Th1 and Th17 responses. Thus, as several T-cell subsets may drive inflammation in a given disease, a broader spectrum of activities targeting possibly all T-cell subsets may be advantageous for a drug.

We demonstrated that a herbal combination is able to significantly suppress the release of proinflammatory cytokines such as IL-1 and TNF-*α*, the general T-helper cell marker IL-2, but also specific T-cell markers IFN-*γ*, IL-5, and IL-17 from nasal polyp tissue upon SEB stimulation, indicating the suppression of all prominent T helper cell populations. The combination of *S. baicalensis* and *E. senticosus* at a concentration of 10 *μ*g/mL resulted in a significant, nearly complete, suppression of Th1 and Th2 cells and a partial suppression of Th-17 cells. The suppressive activity was not inferior and could be considered to be superior to that of an established topical corticosteroid, fluticasone propionate (FP), for IL-1*β*, TNF-*α*, IL-2, IFN-*γ*, IL-5, and IL-17. Furthermore, the herbal product could be shown to be nontoxic to the mucosa, and even mildly stimulating the cilia beat frequency, leading to a faster clearance of the mucociliary transport.

There is evidence from other sources that the single compounds used here do have anti-inflammatory activities. *S. baicalensis* is one of the most popular and multi-purpose herbs used in China traditionally for treatment of inflammation and bacterial and viral infections; it has a strong anti-inflammatory activity and low toxicity. The bioactive components of Scutellaria have been confirmed to be flavones [27]. *Scutellaria baicalensis *Georgi has been used as an antiinflammatory and anti-allergic agent in the treatment of a variety of inflammatory diseases such as bronchitis, asthma, and atopic dermatitis [10, 11].

*E. senticosus*, also known as “Siberian ginseng”, in a combination with other herbs proved superior to conventional antiviral medications for reducing severity and duration of influenza infections in a study by Kulichenko et al. [12]. Recently Jung et al. published findings demonstrating robust reduction of inflammatory parameters, including IL-6, TNF-*α*, neutrophil density, and prostaglandin E2 in a mouse air pouch inflammatory model [13]. Two of the three herbals used by Jung et al. were *S. baicalensis* and *E. senticosus*.

Although vitamin C was not shown to add to the benefit of the herbal compounds in terms of reduction of mediator release from mast cells or cytokine release from T cells, vitamin C has an effect on the ciliary beat frequency. This effect may counteract the reduction of mucociliary clearance in allergic rhinitis and contribute to avoid an impairment of clearance in chronic sinus disease [28].

These findings indicate that the herbal composition may represent a promising therapeutic approach for airway diseases including allergic rhinitis, as it demonstrates a double interference with early-and late-phase reactions (involving mast cells and eosinophils) ex vivo, but also may include acute rhinosinusitis, chronic rhinosinusitis (both involving Th1 cytokines), and nasal polyps (a mostly Th2-driven disease in Caucasians) by its suppressive activity on cytokine release in the human mucosal model. Also, in comparison with topical corticosteroids, the composition is therefore uniquely positioned to act as a topically applied anti-inflammatory agent in nasal allergies, chronic and acute rhinosinusitis, nasal polyp disease, and eventually also lower airway disease such as asthma and COPD. Similarly, it may be beneficial in the therapy of other inflammatory conditions unrelated to the airways, if sufficient concentrations can be achieved via oral route. Our findings merit further evaluation in patient populations, to establish the clinical relevance and the clinical effect of our ex vivo tested formula.

## 5. Conclusions

We show in an ex vivo human nasal mucosal model that a combination of *S. baicalensis*, *E. senticosus,* and vitamin C may be able to significantly block allergic early and late phase mediators and substantially suppress the release of proinflammatory, Th1-, Th2-and Th17- derived cytokines, at least comparable to a topical corticosteroid. These findings support further research with these herbal compounds in clinical studies including allergic rhinitis, and acute and chronic rhinosinusitis.

## Figures and Tables

**Figure 2:**
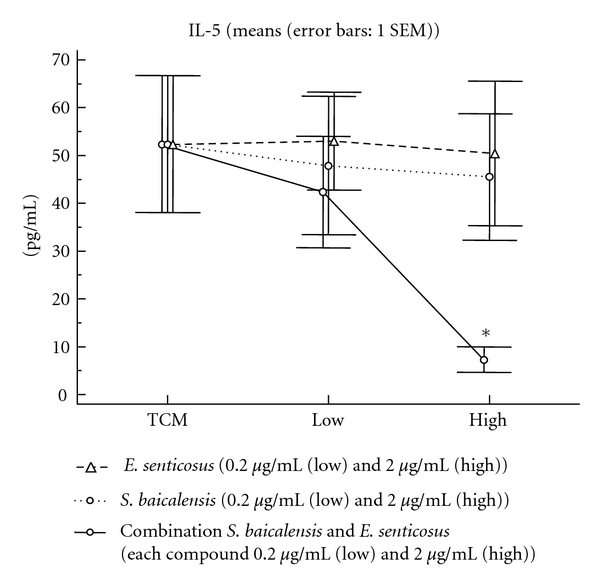
Anti-IgE-induced cytokine release. Anti-IgE stimulation of nasal polyps resulted in an increase in IL-5 within 6 hours. The combination of *S. baicalensis* and *E. senticosus*, but no single component, resulted in a significant inhibition of IL-5 release at a concentration of 2 *μ*g/mL of each compound.

## Abbreviations

AR: Allergic rhinitis
ARS: Acute rhinosinusitis
CRS: Chronic rhinosinusitis
CRSsNP: Chronic rhinosinusitis without nasal polyps
CRSwNP: Chronic rhinosinusitis with nasal polyps
FP: Fluticasone propionate
IFN: Interferon
IL: Interleukin
PGD2: Prostaglandin D2
Th: T helper
TNF: Tumor necrosis factor
*S. baicalensis*: *Scutellaria baicalensis*
*E. senticosus*: *Eleutherococcus senticosus.*
